# Intra- and inter­molecular C—H⋯F hydrogen bonds in the crystal structure of 1,2-bis­[2-(2,3,4,5-tetra­fluoro­phen­yl)ethyn­yl]benzene

**DOI:** 10.1107/S2056989024010995

**Published:** 2024-11-22

**Authors:** Eric Bosch, Nathan P. Bowling

**Affiliations:** a Chemistry and Biochemistry Department Missouri State University, 901 South National Avenue Springfield MO 65897 USA; b Department of Chemistry, University of Wisconsin-Stevens Point, 2101 Fourth Avenue, Stevens Point, WI 54481, USA; Texas A & M University, USA

**Keywords:** crystal structure, C-H⋯F hydrogen bonding, intra­molecular hydrogen bonding

## Abstract

The title mol­ecule, C_22_H_6_F_8_, crystallizes in the monoclinic space group *P*2_1_/*c* with two unique mol­ecules in the asymmetric unit and *Z* = 8. Each mol­ecule features a short intra­molecular *sp*^2^-C—H⋯F hydrogen bond with H⋯F separations at 2.363 (14) and 2.270 (14) Å, corresponding to 91 and 87.5% of the sum of the van der Waals radii, and C—H⋯F angles of 158.3 (14) and 166.8 (14)°, respectively. Each mol­ecule also forms an inter­molecular bifurcated CH⋯(F)_2_ inter­action with H⋯F distances ranging from 2.500 (16) to 2.597 (17) Å.

## Abstract 2

The structure of the aryl ethynylene mol­ecule, 1,2-*bis*-(2,3,4,5-tetra­fluoro­phenyl­ethyn­yl) benzene includes two unique mol­ecules in the unit cell. Both feature intra­molecular *sp*^2^-C—H⋯F hydrogen bonds in addition to inter­molecular C—H⋯F hydrogen bonds.

## 1. Chemical context

Non-covalent inter­actions, including hydrogen bonding, halogen bonding and coordinate bonds, are incorporated in the design and deliberate formation of multicomponent supra­molecular solids with desired physical properties (Panja & Adams, 2021 ▸). Amongst hydrogen bonds, the existence of C—H hydrogen bonds to O and N has long been recognized and extensively reviewed (Desiraju, 1991 ▸). The focus of this report is the lesser-known C—H⋯F hydrogen bond. The role of organic fluorine as a C—H hydrogen-bond acceptor, while controversial, is now accepted to be a weak inter­action (Cole & Taylor, 2022 ▸). The title compound, 1,2-*bis*-(2,3,4,5-tetra­fluoro­phenyl­ethyn­yl) benzene, was specifically prepared to probe intra­molecular C—H⋯F hydrogen bonding. We had previously used the same framework to demonstrate intra­molecular halogen bonding (Widner *et al.*, 2014 ▸) and intra­molecular C—H⋯N hydrogen bonding (Bosch *et al.*, 2015 ▸).
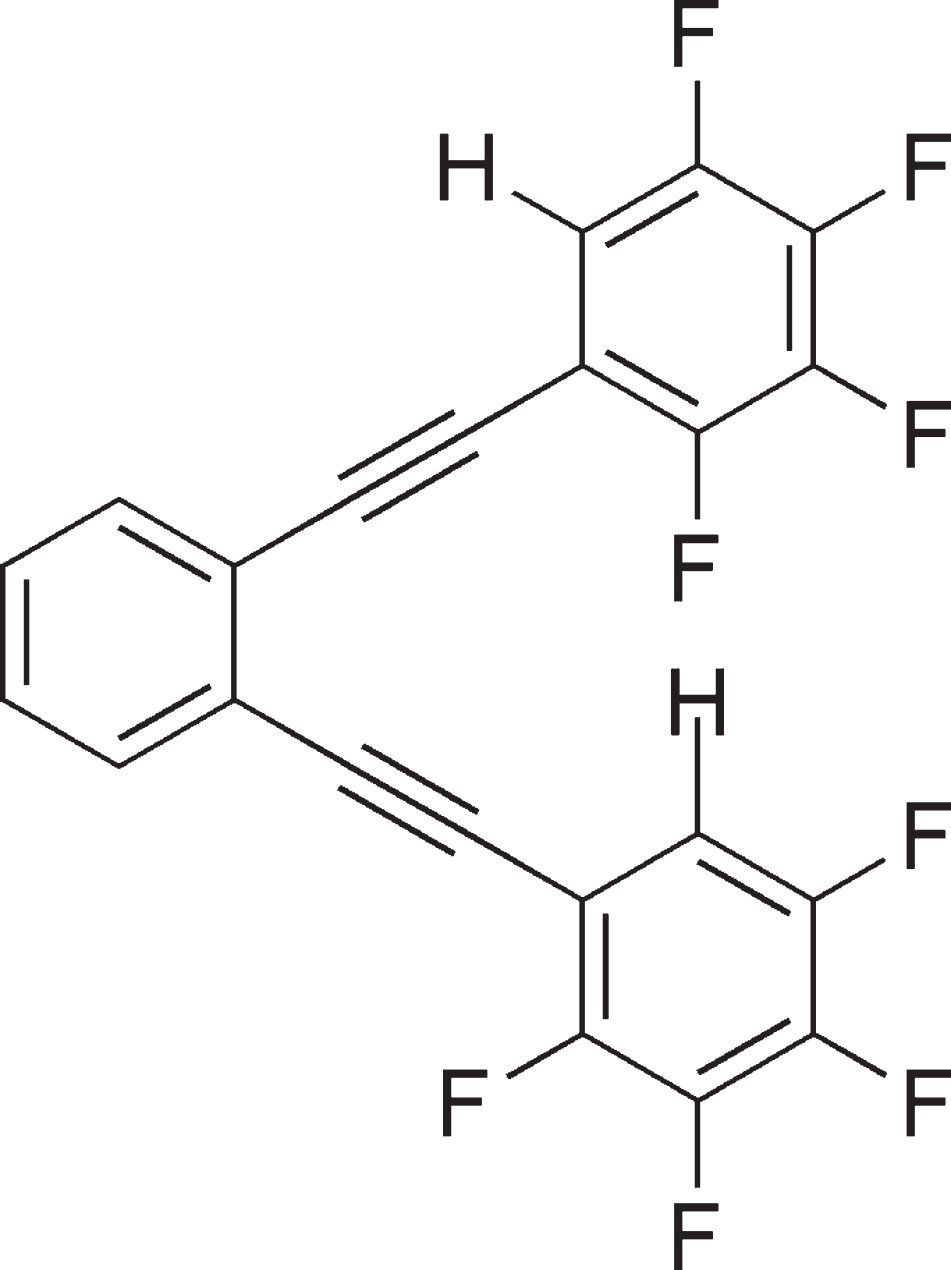


## 2. Structural commentary

**Figure 1:**
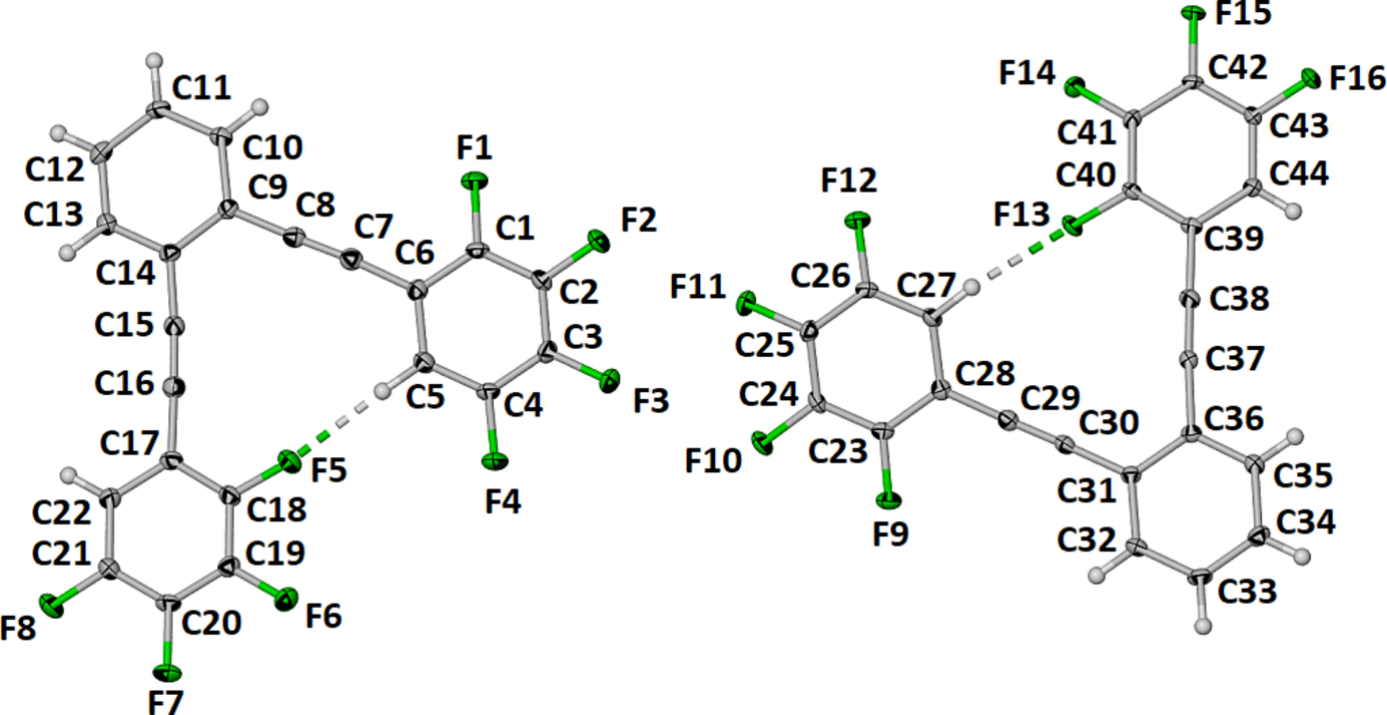
The mol­ecular structure of the title compound with displacement ellipsoids drawn at the 50% probability level and the intra­molecular C—H⋯F hydrogen bonds shown as dashed lines.

The compound C_22_H_6_F_8_ crystallizes in the monoclinic space group *P*2_1_/*c* with two unique mol­ecules in the asymmetric unit. In each mol­ecule, as shown in Fig. 1 ▸, an H atom on one tetra­fluorphenyl ring forms an intra­molecular hydrogen bond to an F atom on the second tetra­fluoro­phenyl ring. The inter­molecular dihedral angle between the two core benzene rings, C1–C6 and C23–C28, is 5.34 (6)°. The intra­molecular C—H⋯F hydrogen bonds (Table 1 ▸) have separations H5⋯F5 and H27⋯F13 of 2.270 (14) and 2.363 (14) Å and C—H⋯F angles of 166.8 (14) and 158.3 (14)°, respectively. These separations are 87.5 and 91.0% of the sum of the van der Waals radii (Bondi, 1964 ▸).

The two unique mol­ecules are essentially planar and coplanar in the crystal structure. The intra­molecularly hydrogen-bonded tetra­fluoro­phenyl rings are slightly twisted with respect the central diethynyl benzene ring. The intra­molecular dihedral angles between tetra­fluoro phenyl rings that are C—H⋯F hydrogen bonded are 13.01 (6)° between benzene rings C1–C6 and C17–C22 and 9.63 (6)° between benzene rings C23–C28 and C39–C44. The F⋯F contacts between the adjacent mol­ecules in the asymmetric unit are 3.0900 (15), 3.1719 (15) and 3.0223 (15)Å for F3⋯F10, F2⋯F11 and F3⋯F11, respectively. These separations are above the sum of the van der Waals radii of 2.80 Å.

## 3. Supra­molecular features

**Figure 2:**
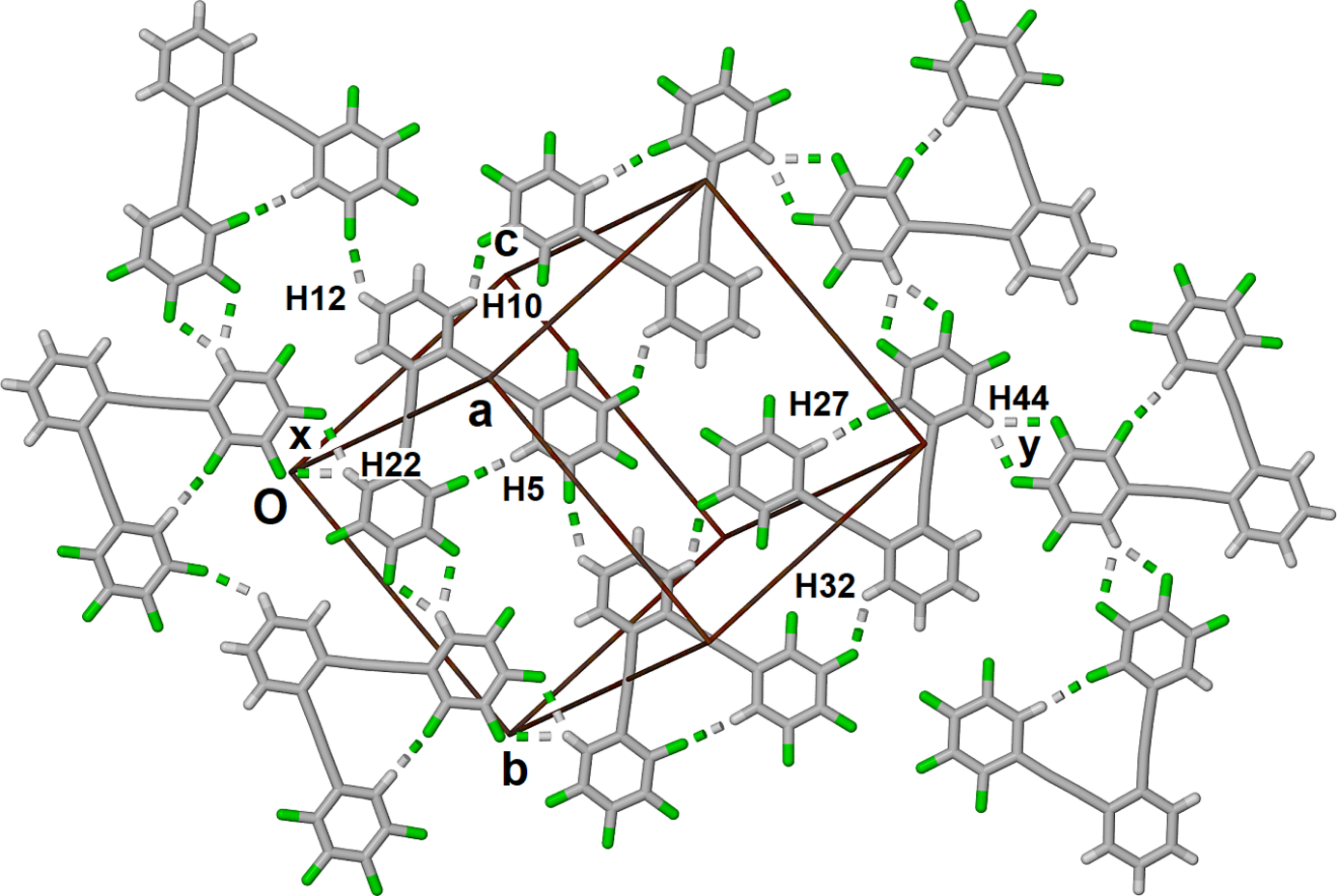
Partial view of one sheet of mol­ecules in the crystal structure with bifurcated C—H⋯F inter­actions labeled ‘x’ and ‘y’.

The two hydrogen atoms on the tetra­fluoro­phenyl rings that are not involved in intra­molecular hydrogen bonding (Table 1 ▸) each have close contacts with two fluorine atoms on adjacent tetra­fluoro­phenyl rings labelled as ‘x’ and ‘y’ in Fig. 2 ▸. The H⋯F separations in ‘x’, H22⋯F6^i^ and H22⋯F7^i^, are 2.597 (17) and 2.500 (16) Å, respectively [symmetry code: (i) 1 − *x*, *y* − 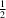, −*z* − 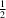] and those in ‘y’, H44⋯F14^ii^ and H44⋯F15^ii^, are 2.574 (16) and 2.460 (15) Å, respectively [symmetry code: (ii) 2 − *x*, 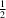 + *y*, 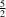 − *z*].

**Figure 3:**
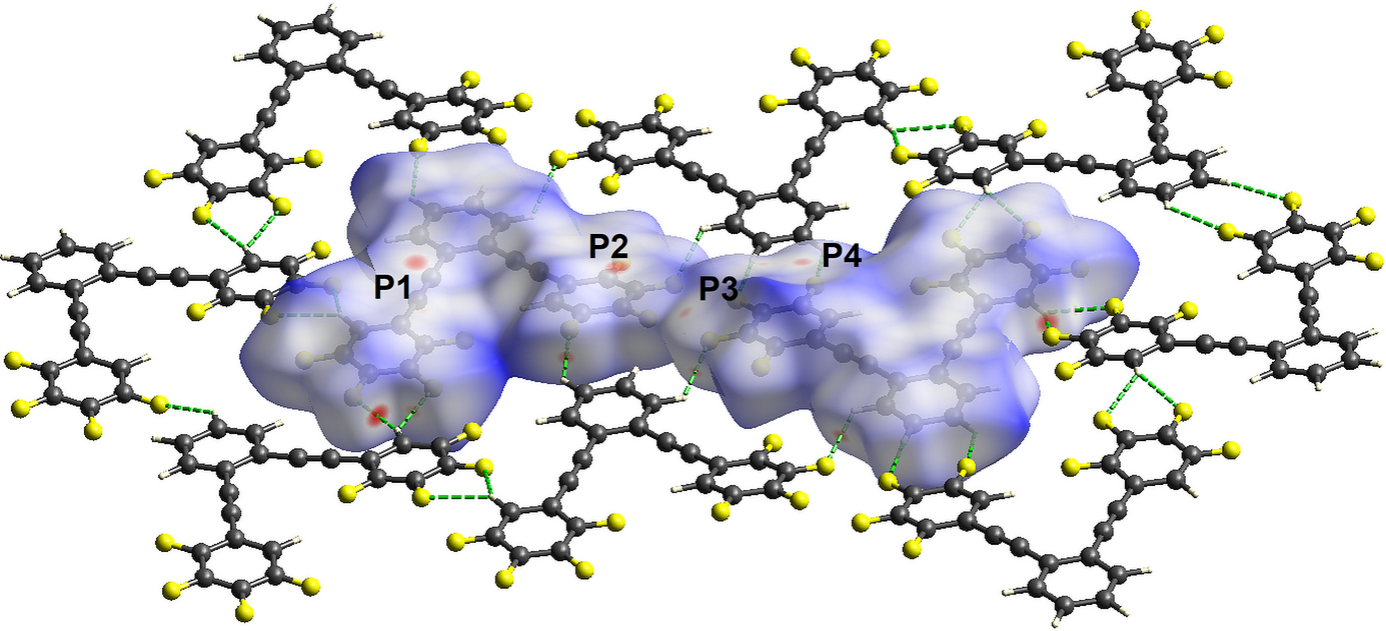
Hirshfeld surface for both mol­ecules in the asymmetric unit with *d*_norm_ mapped over the surface. Included are the mol­ecules within the same plane separated by less than 3.25 Å with C—H⋯F hydrogen bonds shown as green dashed lines. Close contacts related to π-stacked mol­ecules are labelled **P1** to **P4** (see text).

The non-covalent inter­actions were investigated and visualized using Hirshfeld surface analysis (Spackman *et al.*, 2021 ▸) as shown in Fig. 3 ▸. The red spots on the Hirshfeld surface represent close contacts with separations less than the sum of the van der Waals radii while the dark-blue areas correspond to areas where the separations are greater than the sum of the van der Waals radii. The red areas in the plane of the mol­ecules correspond to C—H⋯F hydrogen bonds with the hydrogen-bonded mol­ecules shown.

**Figure 4:**
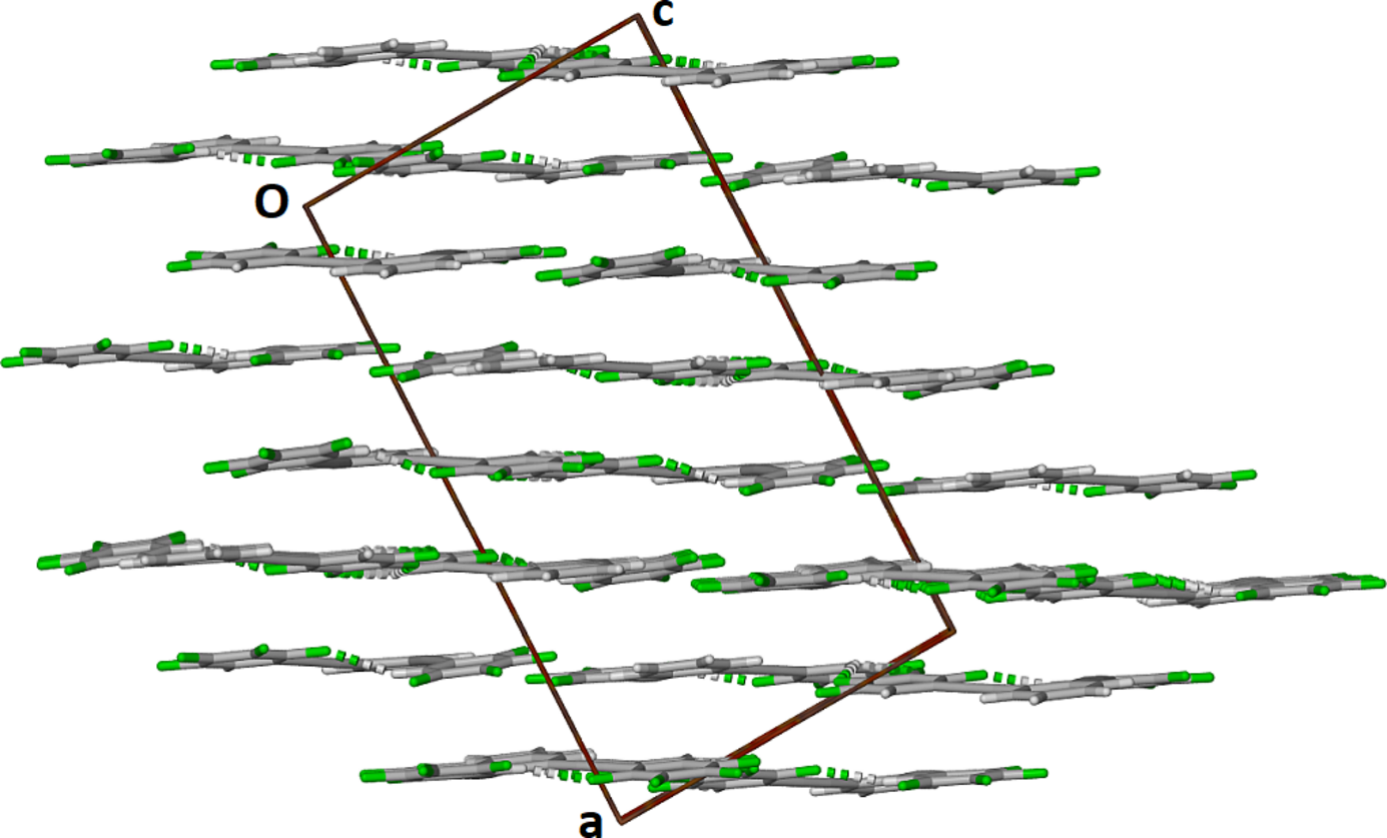
Partial view of the packing within the crystal structure along the *b*-axis.

Also shown in this figure are several close contacts corresponding to π-stacking; labelled **P1**–**P4**. Contacts **P1** and **P2** correspond to inter­actions between an alkynyl C atom and a tetra­fluoro­phenyl C atom. The separations for C38⋯C25^i^ and C25⋯C8^i^ are 3.282 (3) and 3.273 (3) Å respectively [symmetry code: (i) *x*, −*y* + 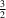, *z* − 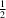]. Contact **P3** is between an F atom and a tetra­fluoro­phenyl H atom while contact **P4** is between two tetra­fluoro phenyl C atoms. The separations are 2.874 (16) and 3.321 (3) Å for F2⋯H5^ii^ and C4⋯C19^ii^, respectively [symmetry code: (ii) *x*, −*y* + 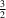, *z* − 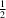]. The planar sheets are π-stacked along the *b*-axis direction as shown in Fig. 4 ▸.

**Figure 5:**
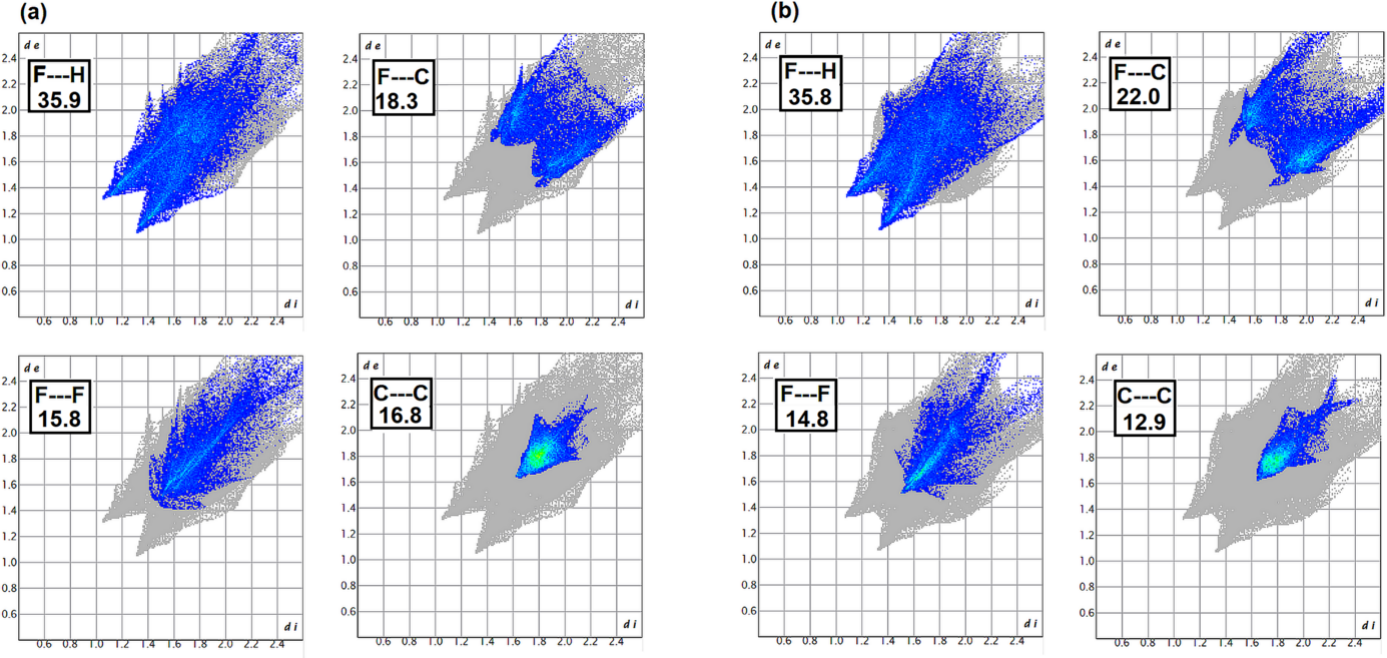
The two-dimensional fingerprint plots delineated into F⋯H contacts, F ⋯C contacts, F⋯F contacts and C⋯C contacts for each of the individual mol­ecules in the asymmetric unit along with the relative percentages. In all cases, reciprocal contacts are included.

The fingerprint plots of the close contacts revealed on the surface of each individual mol­ecule in the asymmetric unit are shown in Fig. 5 ▸ along with the corresponding percentage of the surface area. In both mol­ecules, the dominant inter­action is F⋯H and is largely between adjacent mol­ecules in the same plane. Within each fingerprint plot, the inter­actions with higher frequency are colored turquoise through green, yellow and red with red corresponding to the highest frequency. The C⋯C inter­action that is exclusively correlated to inter­actions between π-stacked mol­ecules has a high frequency of inter­actions focused on distances *d_i_* and *d_e_* of 1.8 Å corresponding to a π–π separation of 3.6 Å, typical for π-stacking.

## 4. Database survey

**Figure 6:**
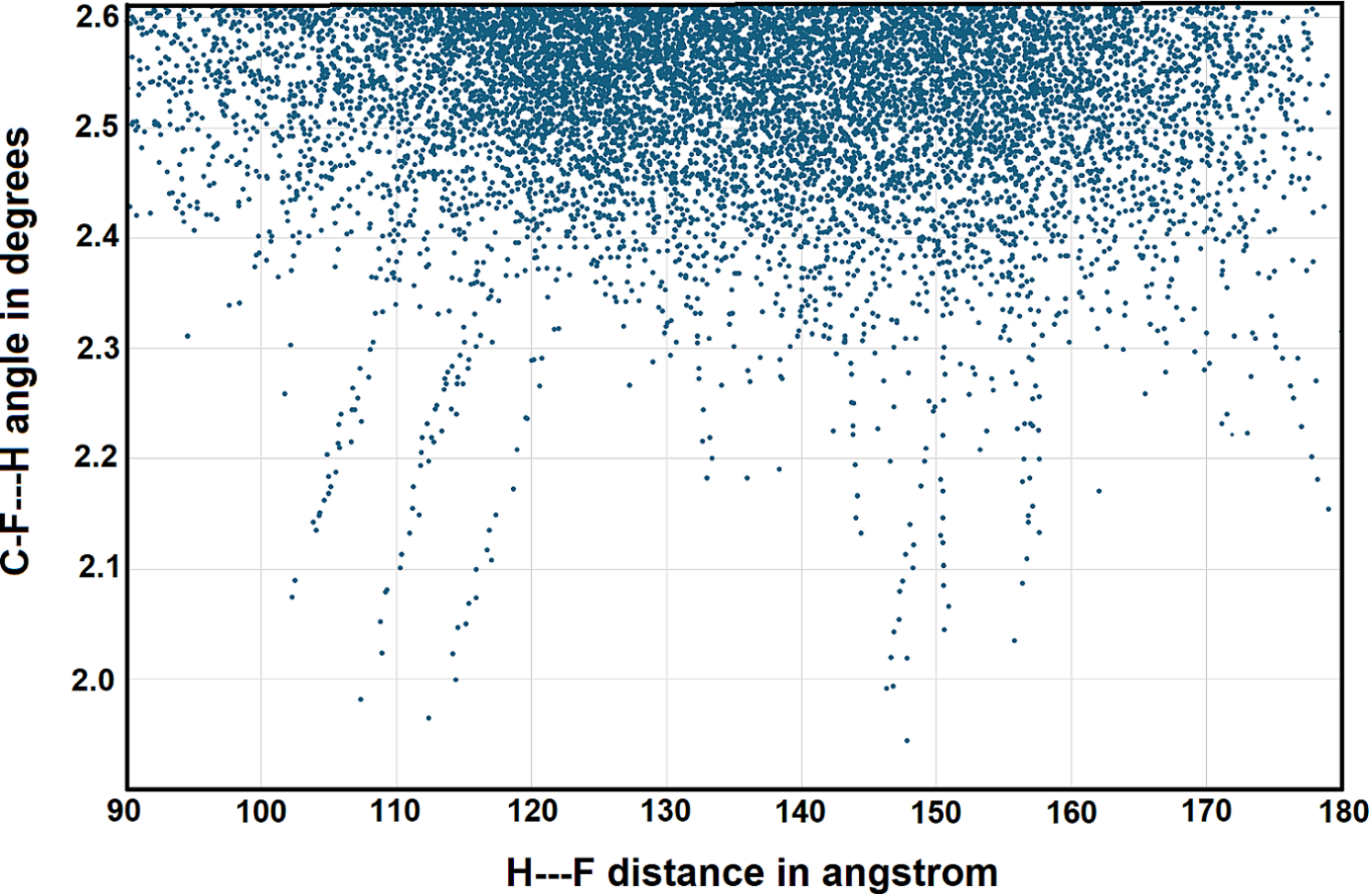
Scatter plot of the hydrogen-bond distance (H⋯F) *versus* the C—F⋯H bond angle for single-crystal X-ray structures deposited in the CSD.

A search of the Cambridge Structural Database (CSD2024.2.0, build 415171; Groom *et al.*, 2016 ▸) for C—H⋯F contacts between an aryl H and an aryl F atom with an H⋯F separation less than or equal to the sum of the van der Waals radii with the C—F⋯H angle between 90 and 180° was performed. The search was further limited to single-crystal structures of organic compounds with 3D coordinates determined, *R* factor less than or equal to 0.1, only non-disordered, non polymeric structures with no errors. This search yielded 7195 unique database entries, including duplicate structures and structures at multiple temperatures, for a total of 13,732 data points. Most of these data points (10,370) have H⋯F separations greater than 2.5 Å, as shown in Fig. 6 ▸. The shorter separations are mostly found within sterically constrained environments. For example, in the structure of 1,2,4-tri­fluoro-3-phenyl­tri­phenyl­ene, an H—F separation of 2.018 Å, 77.6% of the sum of the van der Waals radii, is observed. This inter­action corresponds to a 1,6 H⋯F inter­action between atoms on adjacent phenyl rings in the planar tri­phenyl­ene core of 1,2,4-tri­fluoro-3-phenyl­tri­phenyl­ene (Pan *et al.*, 2023 ▸). This is an example of a sterically induced/enforced short intra­molecular inter­action. Similar, very short inter­molecular separations are reported for co-crystals containing anthracene and octa­fluoro­naphthalene with data collected under pressure (Friedrich *et al.*, 2020 ▸). Thus, at 120 K and atmospheric pressure the shortest C—H⋯F contact is 2.481 Å (Collings *et al.* 2001 ▸), while under a pressure of 22.2 GPa at 293 K this separation is reduced to 1.944 Å.

## 5. Synthesis and crystallization

The title compound, C_22_H_6_F_8_, as prepared by palladium-catalyzed Sonogashira coupling of 1,2,3,4-tetra­fluoro-5-iodo­benzene and 1,2-diethynyl­benzene. 1,2-Diethynyl­benzene was prepared from 1,2-di­iodo­benzene as previously described (Takahashi *et al.*, 1980 ▸) while all other chemicals were commercially available and used as received. 1,2-Diethynyl­benzene (0.22 g, 1.7 mmol) and 1,2,3,4-tetra­fluoro-5-iodo­benzene (0.88 g, 3.8 mmol) were dissolved in NEt3 (4 mL) in a pressure flask. Argon was bubbled through the solution for 10 minutes and PdCl2(PPh3)2 (50 mg, 0.06 mmol) and CuI (25 mg, 0.12 mmol) then added to the flask. The flask was sealed and stirred at 333 K for 24 h. The solvent was evaporated from the crude reaction mixture and the residue purified using flash column chromatography with progressively more polar mixtures of hexane and ethyl acetate to give the title compound as colorless crystals (0.43 g, 59%). Crystals suitable for single-crystal X-ray diffraction were grown on slow evaporation from a di­chloro­methane solution.

## 6. Refinement

Crystal data, data collection and structure refinement details are summarized in Table 2 ▸. H atoms were located in difference maps. H atoms involved in C—H⋯F hydrogen-bonding inter­actions were restrained in the refinement with C—H = 0.95 (2) Å and *U*_iso_(H) = 1.2*U*_eq_(C). All other H atoms were treated as riding atoms in geometrically idealized positions with C—H = 0.95 Å and *U*_iso_(H) = 1.2*U*_eq_(C).

## Supplementary Material

Crystal structure: contains datablock(s) I. DOI: 10.1107/S2056989024010995/jy2054sup1.cif

Structure factors: contains datablock(s) I. DOI: 10.1107/S2056989024010995/jy2054Isup2.hkl

Supporting information file. DOI: 10.1107/S2056989024010995/jy2054Isup3.cdx

Supporting information file. DOI: 10.1107/S2056989024010995/jy2054Isup4.cml

CCDC reference: 2402283

Additional supporting information:  crystallographic information; 3D view; checkCIF report

## Figures and Tables

**Table 1:** Hydrogen-bond geometry (Å, °)

*D*—H⋯*A*	*D*—H	H⋯*A*	*D*⋯*A*	*D*—H⋯*A*
C5—H5⋯F5	0.98 (1)	2.27 (1)	3.230 (2)	167 (1)
C44—H44⋯F14^i^	0.95 (1)	2.57 (2)	3.356 (2)	140 (1)
C44—H44⋯F15^i^	0.95 (1)	2.46 (2)	3.314 (2)	150 (1)
C27—H27⋯F13	0.96 (1)	2.36 (1)	3.278 (2)	158 (1)
C22—H22⋯F6^ii^	0.95 (2)	2.60 (2)	3.389 (2)	141 (2)
C22—H22⋯F7^ii^	0.95 (2)	2.50 (2)	3.345 (2)	148 (2)

Symmetry codes: (i) 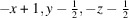; (ii) 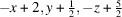.

**Table 2:** Experimental details

Crystal data	
Chemical formula	C_22_H_6_F_8_
*M*_r_	422.27
Crystal system, space group	Monoclinic, *P*2_1_/*c*
Temperature (K)	100
*a*, *b*, *c* (Å)	21.8447 (13), 13.0140 (8), 12.1123 (7)
β (°)	92.620 (1)
*V* (Å^3^)	3439.8 (4)
*Z*	8
Radiation type	Mo *K*α
μ (mm^−1^)	0.16
Crystal size (mm)	0.25 × 0.25 × 0.15

Data collection	
Diffractometer	Bruker APEXII CCD
Absorption correction	Multi-scan (*SADABS*; Krause *et al.*, 2015 ▸)
*T*_min_, *T*_max_	0.672, 0.746
No. of measured, independent and observed [*I* > 2σ(*I*)] reflections	44014, 7632, 5101
*R*_int_	0.048
(sin θ/λ)_max_ (Å^−1^)	0.642

Refinement	
*R*[*F*^2^ > 2σ(*F*^2^)], *wR*(*F*^2^), *S*	0.040, 0.111, 1.01
No. of reflections	7632
No. of parameters	557
No. of restraints	4
H-atom treatment	H atoms treated by a mixture of independent and constrained refinement
Δρ_max_, Δρ_min_ (e Å^−3^)	0.23, −0.29

Computer programs: *APEX2* and *SAINT* (Bruker, 2012 ▸), *SHELXT2018/2* (Sheldrick, 2015*a* ▸), *SHELXL2018/3* (Sheldrick, 2015*b* ▸), and *X-SEED* (Barbour, 2020 ▸).

## supplementary crystallographic information

### 1,2-Bis[2-(2,3,4,5-tetrafluorophenyl)ethynyl]benzene Crystal data

**Table:**

C_22_H_6_F_8_	*F*(000) = 1680
*M**_r_* = 422.27	*D*_x_ = 1.631 Mg m^−^^3^
Monoclinic, *P*2_1_/*c*	Mo *K*α radiation, λ = 0.71073 Å
*a* = 21.8447 (13) Å	Cell parameters from 7323 reflections
*b* = 13.0140 (8) Å	θ = 2.3–27.1°
*c* = 12.1123 (7) Å	µ = 0.16 mm^−^^1^
β = 92.620 (1)°	*T* = 100 K
*V* = 3439.8 (4) Å^3^	Block, colourless
*Z* = 8	0.25 × 0.25 × 0.15 mm

### 1,2-Bis[2-(2,3,4,5-tetrafluorophenyl)ethynyl]benzene Data collection

**Table:**

Bruker APEXII CCD diffractometer	7632 independent reflections
Radiation source: fine-focus sealed tube	5101 reflections with *I* > 2σ(*I*)
Graphite monochromator	*R*_int_ = 0.048
Detector resolution: 8.3660 pixels mm^-1^	θ_max_ = 27.2°, θ_min_ = 1.8°
phi and ω scans	*h* = −28→28
Absorption correction: multi-scan (SADABS; Krause *et al.*, 2015)	*k* = −16→16
*T*_min_ = 0.672, *T*_max_ = 0.746	*l* = −15→15
44014 measured reflections	

### 1,2-Bis[2-(2,3,4,5-tetrafluorophenyl)ethynyl]benzene Refinement

**Table:**

Refinement on *F*^2^	Primary atom site location: dual
Least-squares matrix: full	Secondary atom site location: diffmap
*R*[*F*^2^ > 2σ(*F*^2^)] = 0.040	Hydrogen site location: mixed
*wR*(*F*^2^) = 0.111	H atoms treated by a mixture of independent and constrained refinement
*S* = 1.01	*w* = 1/[σ^2^(*F*_o_^2^) + (0.0515*P*)^2^ + 1.115*P*] where *P* = (*F*_o_^2^ + 2*F*_c_^2^)/3
7632 reflections	(Δ/σ)_max_ = 0.001
557 parameters	Δρ_max_ = 0.23 e Å^−^^3^
4 restraints	Δρ_min_ = −0.29 e Å^−^^3^

### 1,2-Bis[2-(2,3,4,5-tetrafluorophenyl)ethynyl]benzene Special details

**Table:**

Geometry. All esds (except the esd in the dihedral angle between two l.s. planes) are estimated using the full covariance matrix. The cell esds are taken into account individually in the estimation of esds in distances, angles and torsion angles; correlations between esds in cell parameters are only used when they are defined by crystal symmetry. An approximate (isotropic) treatment of cell esds is used for estimating esds involving l.s. planes.

### 1,2-Bis[2-(2,3,4,5-tetrafluorophenyl)ethynyl]benzene Fractional atomic coordinates and isotropic or equivalent isotropic displacement parameters (Å^2^)

**Table:**

	*x*	*y*	*z*	*U*_iso_*/*U*_eq_	
F1	0.77779 (5)	0.93658 (8)	0.51579 (9)	0.0338 (3)	
C1	0.79548 (8)	0.86262 (14)	0.58746 (14)	0.0214 (4)	
F2	0.75740 (5)	0.73905 (9)	0.45920 (8)	0.0314 (3)	
C2	0.78455 (8)	0.76168 (14)	0.55770 (14)	0.0212 (4)	
F3	0.79008 (5)	0.58519 (8)	0.60076 (9)	0.0298 (3)	
C3	0.80089 (8)	0.68326 (14)	0.62972 (15)	0.0218 (4)	
F4	0.84323 (5)	0.63004 (8)	0.80143 (9)	0.0340 (3)	
C4	0.82795 (8)	0.70779 (14)	0.73179 (15)	0.0227 (4)	
F5	0.92433 (5)	0.84712 (8)	0.98359 (9)	0.0322 (3)	
C5	0.83980 (8)	0.80741 (14)	0.76191 (15)	0.0235 (4)	
F6	0.97196 (5)	0.70715 (8)	1.12628 (9)	0.0321 (3)	
C6	0.82350 (8)	0.88797 (14)	0.68959 (14)	0.0207 (4)	
F7	1.01924 (5)	0.76616 (9)	1.32791 (9)	0.0320 (3)	
C7	0.83445 (8)	0.99237 (15)	0.72119 (15)	0.0233 (4)	
F8	1.01963 (5)	0.96706 (9)	1.38461 (9)	0.0367 (3)	
C8	0.84329 (8)	1.07804 (14)	0.75469 (15)	0.0216 (4)	
F9	0.71615 (5)	0.16630 (8)	0.47865 (8)	0.0280 (3)	
C9	0.84968 (8)	1.18143 (13)	0.79439 (14)	0.0192 (4)	
F10	0.74191 (5)	0.36203 (8)	0.53715 (8)	0.0291 (3)	
C10	0.82886 (8)	1.26325 (14)	0.72793 (15)	0.0215 (4)	
H10	0.812171	1.250130	0.655498	0.026*	
F11	0.72140 (5)	0.51762 (8)	0.39096 (9)	0.0273 (3)	
C11	0.83237 (8)	1.36323 (14)	0.76679 (15)	0.0229 (4)	
H11	0.817786	1.418206	0.721180	0.027*	
F12	0.67428 (5)	0.47675 (8)	0.18546 (9)	0.0314 (3)	
C12	0.85710 (8)	1.38342 (14)	0.87206 (15)	0.0245 (4)	
H12	0.859125	1.452112	0.898504	0.029*	
F13	0.57293 (5)	0.25053 (8)	0.01742 (8)	0.0246 (2)	
C13	0.87890 (8)	1.30360 (14)	0.93890 (15)	0.0225 (4)	
H13	0.896161	1.318106	1.010618	0.027*	
F14	0.52464 (5)	0.39200 (8)	−0.12408 (8)	0.0275 (3)	
C14	0.87566 (8)	1.20176 (14)	0.90146 (15)	0.0199 (4)	
F15	0.47673 (5)	0.33454 (8)	−0.32557 (8)	0.0269 (3)	
C15	0.89857 (8)	1.12068 (14)	0.97172 (14)	0.0212 (4)	
F16	0.47389 (5)	0.13386 (8)	−0.38270 (8)	0.0274 (3)	
C16	0.91961 (8)	1.05709 (14)	1.03384 (15)	0.0230 (4)	
C17	0.94577 (8)	0.98316 (14)	1.11011 (15)	0.0210 (4)	
C18	0.94714 (8)	0.87951 (14)	1.08257 (14)	0.0220 (4)	
C19	0.97165 (8)	0.80652 (14)	1.15475 (15)	0.0226 (4)	
C20	0.99584 (8)	0.83679 (14)	1.25661 (15)	0.0234 (4)	
C21	0.99569 (8)	0.94005 (15)	1.28511 (15)	0.0243 (4)	
C22	0.97135 (8)	1.01266 (15)	1.21354 (15)	0.0231 (4)	
C23	0.70518 (8)	0.24157 (14)	0.40448 (14)	0.0192 (4)	
C24	0.71860 (8)	0.34157 (14)	0.43517 (14)	0.0198 (4)	
C25	0.70821 (8)	0.42049 (14)	0.36111 (15)	0.0204 (4)	
C26	0.68407 (8)	0.39796 (14)	0.25635 (14)	0.0205 (4)	
C27	0.67024 (8)	0.29921 (14)	0.22483 (14)	0.0195 (4)	
C28	0.68050 (8)	0.21840 (14)	0.29938 (14)	0.0189 (4)	
C29	0.66737 (8)	0.11407 (14)	0.26837 (14)	0.0204 (4)	
C30	0.65718 (8)	0.02747 (14)	0.23943 (14)	0.0198 (4)	
C31	0.64841 (7)	−0.07709 (13)	0.20536 (14)	0.0177 (4)	
C32	0.66839 (8)	−0.15734 (14)	0.27533 (15)	0.0222 (4)	
H32	0.686437	−0.141961	0.346255	0.027*	
C33	0.66212 (8)	−0.25874 (14)	0.24218 (15)	0.0238 (4)	
H33	0.675455	−0.312447	0.290666	0.029*	
C34	0.63633 (8)	−0.28225 (14)	0.13805 (16)	0.0241 (4)	
H34	0.632677	−0.351878	0.115140	0.029*	
C35	0.61592 (8)	−0.20398 (14)	0.06754 (15)	0.0205 (4)	
H35	0.598433	−0.220417	−0.003519	0.025*	
C36	0.62093 (7)	−0.10131 (13)	0.10035 (14)	0.0179 (4)	
C37	0.59700 (8)	−0.02175 (13)	0.02839 (14)	0.0181 (4)	
C38	0.57445 (8)	0.04127 (14)	−0.03304 (14)	0.0195 (4)	
C39	0.54855 (7)	0.11567 (13)	−0.10834 (14)	0.0176 (4)	
C40	0.54873 (7)	0.21924 (13)	−0.08080 (13)	0.0176 (4)	
C41	0.52474 (8)	0.29280 (13)	−0.15302 (14)	0.0191 (4)	
C42	0.50012 (8)	0.26319 (13)	−0.25523 (14)	0.0195 (4)	
C43	0.49908 (8)	0.16028 (13)	−0.28360 (14)	0.0194 (4)	
C44	0.52294 (8)	0.08682 (14)	−0.21200 (15)	0.0200 (4)	
H5	0.8594 (7)	0.8242 (13)	0.8339 (12)	0.015 (4)*	
H44	0.5228 (8)	0.0162 (11)	−0.2305 (14)	0.018 (5)*	
H27	0.6514 (7)	0.2864 (13)	0.1527 (12)	0.014 (4)*	
H22	0.9727 (8)	1.0835 (12)	1.2336 (16)	0.026 (5)*	

### 1,2-Bis[2-(2,3,4,5-tetrafluorophenyl)ethynyl]benzene Atomic displacement parameters (Å^2^)

**Table:**

	*U*^11^	*U*^22^	*U*^33^	*U*^12^	*U*^13^	*U*^23^
F1	0.0503 (7)	0.0241 (6)	0.0262 (6)	0.0040 (5)	−0.0059 (5)	0.0067 (5)
C1	0.0253 (10)	0.0191 (9)	0.0195 (9)	0.0047 (7)	−0.0006 (7)	0.0053 (7)
F2	0.0432 (7)	0.0315 (6)	0.0186 (6)	0.0005 (5)	−0.0095 (5)	−0.0030 (5)
C2	0.0226 (9)	0.0255 (10)	0.0154 (9)	0.0020 (7)	−0.0022 (7)	−0.0020 (7)
F3	0.0379 (6)	0.0175 (6)	0.0334 (6)	−0.0002 (5)	−0.0056 (5)	−0.0036 (5)
C3	0.0235 (9)	0.0167 (9)	0.0252 (10)	0.0004 (7)	0.0008 (7)	−0.0035 (7)
F4	0.0479 (7)	0.0234 (6)	0.0295 (6)	0.0036 (5)	−0.0115 (5)	0.0081 (5)
C4	0.0266 (10)	0.0186 (10)	0.0226 (9)	0.0046 (7)	−0.0027 (7)	0.0048 (8)
F5	0.0398 (7)	0.0300 (6)	0.0255 (6)	0.0037 (5)	−0.0113 (5)	−0.0024 (5)
C5	0.0250 (10)	0.0238 (10)	0.0212 (10)	0.0022 (8)	−0.0030 (8)	−0.0010 (8)
F6	0.0379 (7)	0.0192 (6)	0.0384 (7)	0.0034 (5)	−0.0070 (5)	−0.0005 (5)
C6	0.0217 (9)	0.0185 (9)	0.0220 (9)	0.0015 (7)	0.0020 (7)	−0.0014 (7)
F7	0.0364 (6)	0.0286 (6)	0.0301 (6)	0.0063 (5)	−0.0084 (5)	0.0103 (5)
C7	0.0233 (10)	0.0234 (10)	0.0231 (10)	0.0021 (7)	0.0007 (7)	0.0001 (8)
F8	0.0482 (7)	0.0361 (7)	0.0244 (6)	0.0038 (6)	−0.0141 (5)	−0.0029 (5)
C8	0.0215 (9)	0.0220 (10)	0.0211 (9)	0.0020 (7)	0.0011 (7)	0.0014 (8)
F9	0.0400 (7)	0.0231 (6)	0.0207 (6)	0.0033 (5)	−0.0024 (5)	0.0060 (4)
C9	0.0175 (8)	0.0174 (9)	0.0227 (9)	−0.0001 (7)	0.0008 (7)	0.0011 (7)
F10	0.0370 (6)	0.0323 (6)	0.0172 (5)	0.0008 (5)	−0.0077 (5)	−0.0052 (5)
C10	0.0204 (9)	0.0237 (10)	0.0203 (9)	0.0008 (7)	−0.0002 (7)	0.0034 (8)
F11	0.0329 (6)	0.0173 (6)	0.0313 (6)	−0.0022 (4)	−0.0020 (5)	−0.0053 (5)
C11	0.0228 (9)	0.0191 (9)	0.0267 (10)	0.0023 (7)	0.0006 (7)	0.0070 (8)
F12	0.0454 (7)	0.0208 (6)	0.0272 (6)	0.0037 (5)	−0.0070 (5)	0.0072 (5)
C12	0.0268 (10)	0.0167 (9)	0.0301 (10)	−0.0009 (7)	0.0034 (8)	0.0010 (8)
F13	0.0309 (6)	0.0263 (6)	0.0157 (5)	0.0022 (4)	−0.0079 (4)	−0.0021 (4)
C13	0.0233 (9)	0.0224 (10)	0.0216 (10)	−0.0013 (7)	−0.0014 (7)	−0.0009 (8)
F14	0.0409 (7)	0.0160 (5)	0.0249 (6)	0.0038 (5)	−0.0063 (5)	−0.0018 (4)
C14	0.0175 (8)	0.0188 (9)	0.0233 (9)	−0.0006 (7)	0.0008 (7)	0.0040 (7)
F15	0.0367 (6)	0.0211 (6)	0.0218 (6)	0.0040 (5)	−0.0093 (5)	0.0074 (4)
C15	0.0211 (9)	0.0211 (10)	0.0212 (9)	0.0003 (7)	−0.0005 (7)	−0.0005 (8)
F16	0.0377 (6)	0.0263 (6)	0.0171 (5)	−0.0037 (5)	−0.0113 (5)	0.0001 (4)
C16	0.0226 (9)	0.0232 (10)	0.0232 (10)	0.0005 (8)	0.0011 (7)	0.0003 (8)
C17	0.0189 (9)	0.0205 (9)	0.0236 (9)	0.0020 (7)	−0.0004 (7)	0.0042 (8)
C18	0.0205 (9)	0.0249 (10)	0.0203 (9)	0.0005 (7)	−0.0031 (7)	0.0017 (8)
C19	0.0217 (9)	0.0177 (9)	0.0283 (10)	0.0031 (7)	−0.0013 (7)	0.0008 (8)
C20	0.0214 (9)	0.0236 (10)	0.0248 (10)	0.0041 (7)	−0.0025 (7)	0.0077 (8)
C21	0.0255 (10)	0.0259 (10)	0.0211 (9)	−0.0003 (8)	−0.0038 (7)	−0.0001 (8)
C22	0.0240 (10)	0.0201 (10)	0.0249 (10)	0.0009 (7)	−0.0011 (8)	0.0010 (8)
C23	0.0198 (9)	0.0202 (10)	0.0176 (9)	0.0037 (7)	0.0010 (7)	0.0030 (7)
C24	0.0193 (9)	0.0251 (10)	0.0145 (8)	0.0005 (7)	−0.0030 (7)	−0.0037 (7)
C25	0.0225 (9)	0.0156 (9)	0.0231 (9)	0.0003 (7)	0.0004 (7)	−0.0035 (7)
C26	0.0225 (9)	0.0201 (9)	0.0188 (9)	0.0032 (7)	−0.0010 (7)	0.0050 (7)
C27	0.0210 (9)	0.0213 (10)	0.0158 (9)	0.0015 (7)	−0.0027 (7)	−0.0010 (7)
C28	0.0179 (8)	0.0203 (9)	0.0185 (9)	0.0010 (7)	0.0020 (7)	−0.0021 (7)
C29	0.0197 (9)	0.0225 (10)	0.0190 (9)	0.0014 (7)	−0.0001 (7)	−0.0005 (8)
C30	0.0195 (9)	0.0231 (10)	0.0163 (9)	0.0006 (7)	−0.0023 (7)	0.0015 (7)
C31	0.0174 (8)	0.0168 (9)	0.0187 (9)	0.0007 (7)	−0.0006 (7)	0.0021 (7)
C32	0.0237 (9)	0.0230 (10)	0.0193 (9)	0.0020 (7)	−0.0047 (7)	0.0032 (8)
C33	0.0237 (9)	0.0197 (10)	0.0276 (10)	0.0024 (7)	−0.0030 (8)	0.0088 (8)
C34	0.0249 (10)	0.0165 (9)	0.0307 (10)	0.0023 (7)	−0.0025 (8)	0.0019 (8)
C35	0.0206 (9)	0.0217 (10)	0.0191 (9)	0.0000 (7)	−0.0025 (7)	0.0002 (7)
C36	0.0166 (8)	0.0182 (9)	0.0190 (9)	−0.0004 (7)	0.0002 (7)	0.0035 (7)
C37	0.0191 (9)	0.0179 (9)	0.0170 (9)	−0.0007 (7)	−0.0016 (7)	−0.0006 (7)
C38	0.0187 (9)	0.0215 (10)	0.0182 (9)	−0.0005 (7)	−0.0007 (7)	0.0012 (7)
C39	0.0148 (8)	0.0211 (9)	0.0170 (9)	0.0001 (7)	0.0002 (7)	0.0047 (7)
C40	0.0187 (9)	0.0208 (9)	0.0130 (8)	0.0004 (7)	−0.0027 (7)	0.0012 (7)
C41	0.0220 (9)	0.0134 (9)	0.0219 (9)	0.0004 (7)	−0.0007 (7)	−0.0004 (7)
C42	0.0227 (9)	0.0180 (9)	0.0173 (9)	0.0011 (7)	−0.0043 (7)	0.0066 (7)
C43	0.0228 (9)	0.0197 (9)	0.0153 (8)	−0.0026 (7)	−0.0044 (7)	0.0002 (7)
C44	0.0227 (9)	0.0174 (9)	0.0195 (9)	−0.0027 (7)	−0.0029 (7)	0.0015 (7)

### 1,2-Bis[2-(2,3,4,5-tetrafluorophenyl)ethynyl]benzene Geometric parameters (Å, º)

**Table:**

F1—C1	1.341 (2)	C16—C17	1.434 (2)
C1—C2	1.380 (3)	C17—C18	1.390 (3)
C1—C6	1.394 (2)	C17—C22	1.402 (3)
F2—C2	1.341 (2)	C18—C19	1.382 (2)
C2—C3	1.379 (3)	C19—C20	1.377 (3)
F3—C3	1.342 (2)	C20—C21	1.387 (3)
C3—C4	1.383 (3)	C21—C22	1.373 (3)
F4—C4	1.349 (2)	C22—H22	0.953 (15)
C4—C5	1.368 (3)	C23—C24	1.381 (2)
F5—C18	1.345 (2)	C23—C28	1.393 (2)
C5—C6	1.402 (3)	C24—C25	1.376 (2)
C5—H5	0.978 (13)	C25—C26	1.383 (2)
F6—C19	1.339 (2)	C26—C27	1.370 (2)
C6—C7	1.429 (3)	C27—C28	1.398 (2)
F7—C20	1.346 (2)	C27—H27	0.963 (13)
C7—C8	1.199 (3)	C28—C29	1.434 (3)
F8—C21	1.339 (2)	C29—C30	1.198 (2)
C8—C9	1.433 (3)	C30—C31	1.432 (2)
F9—C23	1.3433 (19)	C31—C32	1.402 (2)
C9—C10	1.398 (2)	C31—C36	1.417 (2)
C9—C14	1.417 (2)	C32—C33	1.384 (3)
F10—C24	1.3411 (19)	C32—H32	0.9500
C10—C11	1.385 (3)	C33—C34	1.392 (3)
C10—H10	0.9500	C33—H33	0.9500
F11—C25	1.342 (2)	C34—C35	1.389 (2)
C11—C12	1.387 (3)	C34—H34	0.9500
C11—H11	0.9500	C35—C36	1.397 (2)
F12—C26	1.3483 (19)	C35—H35	0.9500
C12—C13	1.388 (3)	C36—C37	1.437 (2)
C12—H12	0.9500	C37—C38	1.198 (2)
F13—C40	1.3429 (19)	C38—C39	1.429 (2)
C13—C14	1.401 (3)	C39—C40	1.388 (2)
C13—H13	0.9500	C39—C44	1.402 (2)
F14—C41	1.338 (2)	C40—C41	1.383 (2)
C14—C15	1.432 (2)	C41—C42	1.382 (2)
F15—C42	1.3451 (19)	C42—C43	1.383 (2)
C15—C16	1.196 (2)	C43—C44	1.377 (2)
F16—C43	1.3422 (19)	C44—H44	0.945 (14)
			
F1—C1—C2	118.22 (16)	F9—C23—C24	118.55 (15)
F1—C1—C6	120.41 (16)	F9—C23—C28	120.21 (16)
C2—C1—C6	121.37 (16)	C24—C23—C28	121.24 (16)
F2—C2—C3	119.45 (16)	F10—C24—C25	119.78 (16)
F2—C2—C1	120.37 (16)	F10—C24—C23	120.13 (16)
C3—C2—C1	120.18 (16)	C25—C24—C23	120.09 (16)
F3—C3—C2	120.09 (16)	F11—C25—C24	120.03 (15)
F3—C3—C4	121.07 (16)	F11—C25—C26	120.99 (16)
C2—C3—C4	118.84 (16)	C24—C25—C26	118.98 (16)
F4—C4—C5	120.36 (16)	F12—C26—C27	120.61 (16)
F4—C4—C3	117.97 (16)	F12—C26—C25	117.76 (16)
C5—C4—C3	121.66 (17)	C27—C26—C25	121.63 (16)
C4—C5—C6	120.17 (17)	C26—C27—C28	119.92 (16)
C4—C5—H5	121.2 (10)	C26—C27—H27	119.7 (11)
C6—C5—H5	118.6 (10)	C28—C27—H27	120.3 (11)
C1—C6—C5	117.78 (16)	C23—C28—C27	118.14 (16)
C1—C6—C7	121.60 (16)	C23—C28—C29	120.59 (16)
C5—C6—C7	120.61 (16)	C27—C28—C29	121.26 (16)
C8—C7—C6	175.7 (2)	C30—C29—C28	178.0 (2)
C7—C8—C9	176.33 (19)	C29—C30—C31	176.97 (19)
C10—C9—C14	119.40 (16)	C32—C31—C36	118.97 (16)
C10—C9—C8	119.84 (16)	C32—C31—C30	119.98 (16)
C14—C9—C8	120.75 (16)	C36—C31—C30	121.03 (15)
C11—C10—C9	120.56 (17)	C33—C32—C31	120.72 (16)
C11—C10—H10	119.7	C33—C32—H32	119.6
C9—C10—H10	119.7	C31—C32—H32	119.6
C10—C11—C12	120.24 (17)	C32—C33—C34	120.19 (16)
C10—C11—H11	119.9	C32—C33—H33	119.9
C12—C11—H11	119.9	C34—C33—H33	119.9
C11—C12—C13	120.22 (17)	C35—C34—C33	120.07 (17)
C11—C12—H12	119.9	C35—C34—H34	120.0
C13—C12—H12	119.9	C33—C34—H34	120.0
C12—C13—C14	120.54 (17)	C34—C35—C36	120.50 (16)
C12—C13—H13	119.7	C34—C35—H35	119.7
C14—C13—H13	119.7	C36—C35—H35	119.7
C13—C14—C9	119.01 (16)	C35—C36—C31	119.51 (15)
C13—C14—C15	119.58 (16)	C35—C36—C37	119.69 (15)
C9—C14—C15	121.41 (16)	C31—C36—C37	120.79 (16)
C16—C15—C14	176.2 (2)	C38—C37—C36	176.52 (19)
C15—C16—C17	178.3 (2)	C37—C38—C39	178.59 (19)
C18—C17—C22	117.91 (16)	C40—C39—C44	118.14 (15)
C18—C17—C16	120.57 (17)	C40—C39—C38	120.52 (15)
C22—C17—C16	121.51 (17)	C44—C39—C38	121.34 (16)
F5—C18—C19	117.74 (16)	F13—C40—C41	118.21 (15)
F5—C18—C17	120.46 (15)	F13—C40—C39	120.25 (15)
C19—C18—C17	121.80 (17)	C41—C40—C39	121.54 (15)
F6—C19—C20	120.05 (16)	F14—C41—C42	119.90 (15)
F6—C19—C18	120.49 (16)	F14—C41—C40	120.48 (15)
C20—C19—C18	119.46 (17)	C42—C41—C40	119.61 (16)
F7—C20—C19	119.90 (16)	F15—C42—C41	119.73 (15)
F7—C20—C21	120.46 (16)	F15—C42—C43	120.66 (15)
C19—C20—C21	119.65 (16)	C41—C42—C43	119.60 (15)
F8—C21—C22	120.78 (17)	F16—C43—C44	120.88 (16)
F8—C21—C20	118.23 (16)	F16—C43—C42	118.15 (15)
C22—C21—C20	120.98 (17)	C44—C43—C42	120.97 (16)
C21—C22—C17	120.18 (18)	C43—C44—C39	120.13 (17)
C21—C22—H22	119.9 (12)	C43—C44—H44	121.9 (11)
C17—C22—H22	119.9 (12)	C39—C44—H44	118.0 (11)

### 1,2-Bis[2-(2,3,4,5-tetrafluorophenyl)ethynyl]benzene Hydrogen-bond geometry (Å, º)

**Table:**

*D*—H···*A*	*D*—H	H···*A*	*D*···*A*	*D*—H···*A*
C5—H5···F5	0.98 (1)	2.27 (1)	3.230 (2)	167 (1)
C44—H44···F14^i^	0.95 (1)	2.57 (2)	3.356 (2)	140 (1)
C44—H44···F15^i^	0.95 (1)	2.46 (2)	3.314 (2)	150 (1)
C27—H27···F13	0.96 (1)	2.36 (1)	3.278 (2)	158 (1)
C22—H22···F6^ii^	0.95 (2)	2.60 (2)	3.389 (2)	141 (2)
C22—H22···F7^ii^	0.95 (2)	2.50 (2)	3.345 (2)	148 (2)

Symmetry codes: (i) −*x*+1, *y*−1/2, −*z*−1/2; (ii) −*x*+2, *y*+1/2, −*z*+5/2.
